# On the Diseases and Derangements of the Nervous System, in Their Primary Forms and in Their Modifications, &c.

**Published:** 1841-10-01

**Authors:** 


### Medico-Chirurgical Review,

### No. LXX.

**[No. 30 of a Decennial Series.]**

JULY 1, TO OCTOBER 1, 1841.

### ON THE DISEASES AND DERANGEMENTS OF THE NERVOUS SYSTEM, IN THEIR PRIMARY FORMS AND IN THEIR MODIFICATIONS BY AGE, SEX, CONSTITUTION, HEREDITARY PREDISPOSITION, EXCESSES, GENERAL DISORDER, AND ORGANIC DISEASE. By *Marshall Hall*, M.D. F.R.S. L. & E. &c. &c. &c. Octavo, pp. 380. London, Baillière. 1841.

WE are not sure that Dr. Marshall Hall is well advised in dwelling so much on the injuries he has received. He places himself in the same category with Harvey and with Galileo, and exposes himself to the sneers of those who are inclined (and they are too many) to derogate from his real merits, and disparage his labours and their fruits. The proverbial irritability of authors is displayed by him too prominently. The public either laughs at incessant complaints, or gets tired of them. Controversy and controversialist are both voted a bore, and there is a greater danger impending than opposition—neglect.

Dr. Hall too, overrates the wrongs he has encountered. New views are at first ill understood, and require opposition for their perfect development. Men's minds are too entrenched in old opinions to be taken by storm even by truth. The process of conviction is a slow and a sure one. All this a philosopher should reckon on, and not lose his temper at every doubt and every objection. We conceive that if Dr. Hall had engaged less in altercation, and prosecuted his researches with more equanimity, he would have escaped many annoyances, and stood in just as advanced a position as he occupies at present. He may think us impertinent for offering advice, and yet we *would advise* him to drop the tone of disputation altogether, avoid noticing as well as indulging in personal attacks, and content himself with working out his own discoveries and views.

We do not propose recording those discoveries, nor entering at length upon those views at this time. We have devoted so many pages to them lately, that that would be supererogation. The application, however, of those views to pathology, an application made freely in the work before us, may present some points of novelty and of instruction, and to that application we shall confine ourselves. An article of this description must, of necessity, be desultory.

Dr. Hall has satisfied himself of the seat of the soul and of its properties, and hits materialism a mortal blow. "The principle of action," says he, "in the cerebral system, is the ψυχη, or the immortal *soul*. Upon the cerebrum the soul sits enthroned, receiving the ambassadors, as it were, *from* without, along the *sentient nerves;* deliberating and willing; and sending forth its emissaries and plenipotentiaries, which convey its sovereign mandates, along the *voluntary nerves, to* muscles subdued to volition."

"The acts of the soul are, as I have stated, frequently spontaneous. It is this very character of spontaneous action, indeed, which proves the soul to be free in its agency, independent of organisation (although for a time connected with it, and influenced and manifested by it), and therefore indestructible and immortal. All the acts of mere organic life are the result, the effect, of *physical* stimulus; let this stimulus cease, and its effect ceases—it is death. The functions, the operations of the soul alone are free, spontaneous, unexcited. This is the true argument against *materialism;* but I reserve the task of unfolding it to another and more appropriate opportunity." 4.

We could have wished that Dr. Hall had been more communicative, for his promise to prove all by-and-by is tantalising. As the matter stands, it looks as if the act of *volition* constituted the evidence, and was the office of the *soul*. But if this be so, all animals possessed of *volition* have souls. It would therefore be well to understand exactly what Dr. Hall intends by a soul. We confess that we are of those who believe in the existence of an immortal soul in *man*. But we are more inclined to look to inspired Writ than to physiology for the assurance of it. Physiology, indeed, leaves such high questions in the dark. The sciences do not rise to these arcana of Nature, and what we know we must submissively receive from the lips of the Deity himself.

But to proceed. The following passage is a fair sample of Dr. Hall's style. Its tone will, perhaps, afford a key to much of the acrimonious opposition he has met with.

"It is very different in regard to the true spinal system; in this, the actions are always *excited*. The principle of motion is the *vis nervosa* of Haller, acting in modes and directions unknown to physiologists before the publication of my inquiries, the discovery of which I regard as having laid the foundation of all accurate knowledge of this system, with its extensive applications to physiology and pathology. Indeed, I may assert that the *vis nervosa,* in its *reflected* and *direct* operations, is to the nervous, what the blood, in its *circulation,* is to the vascular system; the former is the *dynamic,* the latter is the *material,* in all the corporeal functions in the animal œconomy, as distinguished from the psychical. Our knowledge of the distinct phenomena of the subdivisions of the nervous system is, further, an important guide in the diagnosis and prognosis of the *diseases* of this system; it is, to the diseases of the brain, the true spinal marrow, the nerves, and the ganglionic system, what auscultation is to those of the viscera within the thorax." 4.

This, at all events, is not calculated to disarm hostility. Dr. Hall presents us with a tabular view of his division of the nervous system, which may be usefully conned. Here it is.

TABLE OF THE NERVOUS SYSTEM.

I. *The entire Nervous System is divisible into—*
- I. *The Cerebral.*
- II. *The True-Spinal.*
- III. *The Ganglionic.*

II. *The Cerebral System is*
- I. *The Seat of the ψυχη, or soul, and*
- II. *The System of*
  - 1. *Sensation and of the Senses.*
  - 2. *Volition and of Spontaneous Motion.*
  - 3. *Sleep and Fatigue.*

III. *The True Spinal System.*
- I. *The Principle of Action is the Vis Nervosa.*
- II. *Its Modes of Action are excited and reflex or direct.*
- III. *The Reflex Functions are those of*
  - 1. *Ingestion and Retention.*
  - 2. *Egestion and Exclusion.*
- IV. *The Direct Functions are*
  - 1. *The Tone* } *of the Muscular Fibre.*
  - 2. *The Irritability* }

IV. *The Ganglionic System.*
- I. *The Principle of Action is the Vis Nervosa.*
- II. *The Mode of Action excited, direct or immediate.*
- III. *The Functions those of*
  - 1. *The Internal Muscular Organs.*
    - 1. *The Heart and Arteries.*
    - 2. *The Stomach and Intestines.*
  - 2. *Nutrition.*
  - 3. *Secretion, &c.*

V. *The System of the Emotions; these are*
- I. *Psychical Affections, acting through*
- II. *The True Spinal, and*
- III. *The Ganglionic Systems.*

*Mixed Functions.* (bracketing II. 1–3 and III. I–II)

*Mixed, in the Œsophagus; the Rectum?* (bracketing III. III to IV. III. 1)

Chapter I. is devoted to the *Anatomy, Physiology, and Pathology of the Cerebral System.*

The Anatomy and Physiology we shall pass over, but pause at the Pathology. Let Dr. Hall apply his views to this.

The cerebral system being the system of the sensations, of judgment, of volition, it is to it that we must refer all morbid conditions of these mental acts or functions. Every derangement of the senses, every form of delirium or of coma, or of perverted imagination or judgment, every act of violence, must be referred to the condition, primary or secondary, of the cerebrum, or cerebellum. The experiments of Majendie and Flourens have shewn that it is impossible, by lacerations or other modes of injury of the *cerebrum* or *cerebellum,* to induce either pain, or contraction in the muscular system. These organs are not endued with *sensibility*, or with the *vis nervosa* of Haller.

When the cerebrum is irritated, delirium ensues. When compressed, coma is induced. When lacerated, we have paralysis of *voluntary* motion. If other phenomena are seen in diseases of the encephalon, they arise from the extension of the influence of these to the true spinal and ganglionic systems, through *irritation* or *pressure*, *counter-irritation* or *counter-pressure*.

The olfactory, the optic, the acoustic nerves are, equally with the cerebrum and cerebellum, incapable of *pain*, or of *exciting movements* in the muscular system, when punctured or lacerated. But when the optic nerve is inflamed or irritated, there is impatience of light; when the membranes of the encephalon are inflamed and the cerebrum irritated, there is delirium. When these several textures are compressed, there is amaurosis, and coma respectively.

It must not be lost sight of, that, not only undue arterial action, and venous congestion, induce morbid states of the cerebral functions, but the state of exhaustion from the loss of blood, the anæmious condition in chlorosis, &c. induce *similar* effects, and present to the physician anxious cases, which frequently try his skill in diagnosis. Too great action, then, of the minute arteries, congestion in the veins, an anæmious state of the vascular system of the encephalon, alike induce morbidly exalted and impaired conditions of the mental and cerebral functions: spectra, delirium, insomnia; amaurosis, stupor, coma; violent voluntary actions, or paralysis of the voluntary motions: these are the symptoms which arise out of these morbid conditions of the cerebral system and functions; and these only. Spasmodic actions depend upon the fact of another system being implicated.

Speaking of paralysis of the face, Dr. Hall makes this remark:—In paralysis of the face, from disease of the opposite hemisphere, the eye-lid can be closed, as represented in *Plate* vii. *fig*. 1; in paralysis of the facial nerve, the orbicularis is paralysed, as we observe in *Plate* vii. *fig*. 2. What is the rationale of this difference? The seventh, like the fifth, is a compound nerve. As the latter embraces excitor and ganglionic filaments, which are not involved in the attack of hemiplegia, so the former comprises a branch belonging to the excito-motory system, which is not affected in disease of the cerebrum.

Both the fifth and the seventh pairs of nerves are, then, more complex than they are represented to be by Sir Charles Bell. The former includes excitor and nutrient nerves, with the nerve of sensation; and it has appropriate origins, distributions, and offices: of its offices, sensation alone is impaired by cerebral disease; but *all* are annihilated by the pressure of a tumour within the cranium. The seventh comprises pure cerebral and and true spinal nerves; the cerebral only is affected in hemiplegia, and the orbicularis retains its power; all are paralysed by the pressure of a tumor below the ear, and we have paralysis of the sphincter of the eye-lid. This remark leads me so observe that *ptosis* is a cerebral paralysis, whilst *lagophthalmia* is one of the true spinal system: *strabismus* may be spasmodic or paralytic and belong to either.

Dr. Hall notices, of course, the fact, that disease confined to one hemisphere of the cerebrum, or of the cerebellum, and to one side of the mesial plane in the tuber annulare, constantly affects the *opposite* side,—whilst disease, confined to one of the lateral columns of the medulla oblongata and medulla spinalis, affects the *corresponding* side, of the muscular system. The encephalon has a *crossed effect;* the medulla spinalis a *direct effect.*

It has been further ascertained that, *in experiments,* lesions of the encephalon induce *paralysis only,* whilst lesions of the medulla oblongata and spinalis induce *convulsion* or *paralysis,* according to its severity.

Some other more apocryphal facts in relation to lesions of certain parts of the brain we pass over. But it may be remarked that in those cases in which hæmorrhagy occupies an extensive space, affecting both hemispheres of the cerebrum,—as in meningeal hæmorrhagy at the summit, or at the base of the brain, in extensive hæmorrhagy within the brain, extending from one hemisphere to the other, or into both ventricles,—*general* paralysis is observed; the same event takes place in the cases in which a clot is formed in the mesial line in the tuber annulare,—the nodus encephali, as it has been termed.

The second chapter is devoted to the *True Spinal or Excito-Motory System.*

Its principle of action, the *vis nervosa* of Haller, and its anatomy, are first dwelt on. A Table of the Anatomy will place it conveniently before the eye.

TABLE OF THE ANATOMY OF THE TRUE SPINAL SYSTEM.

I. *The Incident Motor Branches.*

- 1. *The Trifacial, arising from—*

  - 1. *The Eye-lashes.*

  - 2. *The Alæ Nasi.*

  - 3. *The Nostril.*

  - 4. *The Fauces.*

  - 5. *The Face.*

- 2. *The Pneumogastric, from*

  - 1. *The Pharynx.*

  - 2. *The Larynx.*

  - 3. *The Bronchia.*

  - 4. *The Cardia, Kidney, and Liver.*

- 3. *The Glosso Pharyngeal?*

- 4. *The Posterior Spinal, arising from—*

  - 1. *The General Surface.*

  - 2. *The Glans Penis vel Clitoridis.*

  - 3. *The Anus.*

  - 4. *The Cervix Vesicæ.*

  - 5. *The Cervix Uteri.*

II. *The True Medulla Oblongata and Medulla Spinalis, the Centre of the System.*

III. *The Reflex, Motor Branches.*
- 1. *The Trochlearis* } *Oculi.*
- 2. *The Abducens* } *Oculi.*
- 3. *The Minor Portion of the Fifth.*
- 4. *The Facial, distributed to*
  - 1. *The Orbicularis.*
  - 2. *The Levator Alæ Nasi.*
- 5. *The Pneumogastric or its Accessory.*
  - 1. *The Pharyngeal.*
  - 2. *The Œsophageal and Cardiac.*
  - 3. *The Laryngeal.*
  - 4. *The Bronchial, &c.*
- 6. *The Myo-glossal.*
- 7. *The Spinal Accessory.*
- 8. *The Spinal, distributed to the*
  - 1. *Diaphragm, and to*
  - 2. *The Intercostal and* } *Muscles.*
  - 3. *The Abdominal* } *Muscles.*
- 9. *The Sacral, distributed to*
  - 1. *The Sphincters.*
  - 2. *The Expulsors, the Ejaculators, the Fallopian Tubes, the Uterus, &c.*

And passing to the physiology, Dr. Hall favours us with a similar Table of *it.* They should be placed side by side.

### TABLE OF THE PHYSIOLOGY OF THE TRUE SPINAL SYSTEM.

I. *The Excited Action—*
- 1. *Of the Eyes, the Eye-lids; (and of the Iris?)*
- 2. *Of the Orifices.* { 1. *The Larynx.* 2. *The Pharynx.* }
- 3. *Of the Ingestion.*
  - 1. *Of the Food.*
    - 1. *In Suction;*
    - 2. *In Deglutition.*
  - 2. *Of the Air, or Respiration.*
  - 3. *Of the Semen, or Conception.*
- 4. *Of Exclusion.*
- 5. *Of the Expulsors, or of Egestion.*
  - 1. *Of the Fæces;*
  - 2. *Of the Urine;*
  - 3. *Of the Perspiration;*
  - 4. *Of the Semen;*
  - 5. *Of the Fœtus, or Parturition.*
- 6. *Of the Sphincters.*
  - 1. *The Cardia.*
  - 2. *The Valvula Coli?*
  - 3. *The Sphincter Ani.*
  - 4. *The Sphincter Vesicæ.*

II. *The Direct Action or Influence—*

- I. *In the Tone,* } *of the Muscular System.*

- II. *In the Irritability,* }

Dr. Hall observes that the true spinal marrow may be correctly viewed as a distinct *organ,* totally different in its properties and functions from the cerebrum, and as the centre of a distinct system of nerves, different from the sentient and voluntary, and possessed of a peculiar and special motor power, which acts in incident as well as reflex directions, for special purposes—judging entirely from *experiment.* It may be received as a principle, that every part of the nervous system, which is endowed with the excito-motory power, belongs to this system, whether this power be exerted in the direction of the nerves *from* or *towards* the nervous centres.

He observes, too, that each nerve of the excito-motory system, with the exception, perhaps, of the pneumogastric, is a compound nerve, having a cerebral as well as a true spinal origin. Whether these may be traced by the scalpel is another matter.

On stimulating given incident nerves, the most usual effect produced is a motion of the limbs. But in other instances we have acts of inspiration, of deglutition, of expulsion,—of closure in the eye-lids, larynx, pharynx, and the sphincters. These excitor nerves may be viewed as *guards* of the orifices and exits of the animal frame, as seen in the subsequent *Table:*

### Table of the Guards of the Orifices.

I. *The Trifacial guards—*
1. *The Eye.*
2. *The Nostril—the Ear in the Cetacea,*
3. *The Fauces.*

II. *The Pneumogastric guards—*
1. *The Larynx, the Bronchia.*
2. *The Pharynx, the Cardia.*
3. *The Ureter, the Gall-duct.*

III. *The Spinal Nerves guard—*
1. *The Rectum.*
2. *The Bladder.*
3. *The Vesiculæ Seminales.*
4. *The Uterus.*

With each part of this series of excitor nerves are connected, through the spinal marrow, a corresponding set of motor nerves.

We are tempted to notice Dr. Hall's explanation of the physiological act of respiration. It is the opinion of several physiologists that respiration depends on the medulla oblongata as its primum mobile. It is founded upon the facts that the cerebrum may be removed from above downwards, and the spinal marrow from below upwards, without suspending the acts of inspiration, if the medulla oblongata at the point of origin of the pneumogastric be preserved entire. But if the nerves of the eighth pair be cut, respiration, though difficult, continues. "From these several experiments," says Dr. Hall, "we should be apt to conclude, that neither the cerebrum nor the pneumogastric nerves are necessary for the acts of inspiration, since these acts are renewed when either is removed. The truth, however, is, that although the acts of inspiration continue without *either*, they will not continue without *both*. *Each* may be removed *singly*; but *if both* be removed, the inspirations cease, as in the experiment of dividing the medulla oblongata at the origin of the pneumogastric nerves, an experiment hitherto unexplained. In fact, inspiration may be a voluntary act, induced by the agency of the cerebrum, the pneumogastric nerves being divided; or it may be an excited act, excited through the agency of the pneumogastric nerves, the cerebrum being removed. If, in this latter circumstance, the pneumogastric nerves be divided, the acts of inspiration cease! In this last fact we have, then, the proof that the medulla oblongata is not the primum mobile of respiration; but that the pneumogastric nerve is that primum mobile, as an excitor of respiration, essential and necessary when the agency of volition, with its organ, the cerebrum, is removed,—an important conclusion, by which many difficulties, and an interesting question, are at once solved."

Dr. Hall considers the carbonic acid evolved in the lungs, as the stimulus or exciting cause of inspiration. And he ingeniously explains in this manner the accordance between respiration and circulation. This proportion, he remarks, obtains from the highest degree of activity in an animal, even to the most complete quiescence in hybernation. The evolution of carbonic acid is greater in proportion to the rapidity of the circulation: this carbonic acid is itself the exciting cause of inspiration; this act will, therefore, be repeated more or less frequently as the circulation, and with it the evolution of carbonic acid, is more or less rapid.

But the pneumo-gastric is not the sole excitor of inspiration. There are several, arranged thus:—

1. *The Trifacial.*
2. *The Pneumogastric.*
3. *The Spinal.*

But if these constitute the *excitor* nerves of inspiration, the medulla oblongata must be viewed as the organ which *combines* the various muscles into a system, and the various nerves comprised in the respiratory system of Sir Charles Bell are the true *motor* nerves of respiration.

Speaking of the *excited* act of deglutition, Dr. Hall alludes to a well known statement of Mr. Mayo's, viz.—If the action of deglutition be voluntarily performed several times in succession, and nothing but saliva swallowed, the parts become *fatigued*, and the operation cannot be immediately repeated. The real explanation of this last singular and interesting fact is this: an excited act requires a stimulus or excitor; the saliva is that stimulus in the first and second acts of deglutition; but in a third, attempted promptly after the second, this stimulus is wanting; the act consequently fails for want of its excitor.

*Action of the Cardia.*—This is paralysed on dividing the pneumo-gastric nerves. Dr. Hall states, that there seems to be a property of actual, not to say *active*, dilatation, in the cardia, as well as in some other sphincters. If we irritate the fauces, and induce the first disposition to vomit, we may be perfectly conscious of an act of opening of the cardia; a portion of gas escapes and ascends the œsophagus. A *similar* event doubtless takes place during deglutition. In regard to the sphincters, it is plain that they have the *power* of dilatation: gas may escape from the intestine noiselessly, and the urine from the bladder without effort; events very different from that generally supposed, of the sphincters being overcome by an antagonist force. There is a fact of *practical* interest connected with this view; sometimes hæmorrhoids are protruded so that no force of pressure can replace them; but, by inducing the patient to make the effort usual in voiding the intestine, the sphincter relaxes positively, and the hæmorrhoidal tumor is returned with facility by gentle pressure.

An interesting Section is made up of *Practical Applications of the Physiological Principles laid down.*

1. *Treatment of Choking.*—The danger from a morsel of food remaining in the pharynx arises from a reflex action closing the glottis. Dr. H. proposes this immediate remedy.

Pressure being made on the abdomen, to prevent the descent of the diaphragm, a forcible blow should be made by the flat hand on the thorax. The effect of this is to induce an effort *similar* to that of expiration; the larynx being closed, œsophageal vomiting takes place, and the morsel is dislodged.

If this plan fail, not an instant being lost, the pressure should be kept up on the abdomen, the finger should be introduced into the throat, and the same smart and forcible blow made on the thorax as before. By the irritation of the fauces, the cardia is opened, and, by the blow on the thorax, (firm pressure being made on the abdomen), an effort similar to that of expiration, with a closed larynx, is made, and a direct vomiting occurs, and the morsel of food is carried away.

2. *Mode of passing the Stomach Tube.*—It is often required to pass the *stomach-tube* in cases of *poisoning*. Now, in doing this, the great object is to avoid the *excitors of vomiting*. This is done by passing the tube boldly through the isthmus faucium,—avoiding touching the base of the tongue, the posterior gums, or the veil of the palate,—to the posterior part of the pharynx. In this last part there are no nerves but those of the excitors and motors of deglutition; so that there is no difficulty in passing the stomach-tube, with these precautions, freely on into the œsophagus, and finally into the stomach itself. We cannot say that our experience has quite borne this out. Irresistible efforts to vomit are not unfrequently excited when the tube has passed some way down the œsophagus.

3. *The Use of the Pharyngeal Tube.*—It has been usual, with the object of administering food, in cases of cut-throat, of mania, &c. also to introduce the tube through the whole course of the œsophagus into the stomach itself. It occurred to Dr. Hall that it was unnecessary to adopt this measure, and that to introduce a tube, which may be called the *pharyngeal-tube*, within the influence of the excitor nerves of deglutition, and within the grasp of the constrictors of the pharynx, would be sufficient. The interesting experiment was performed by Mr. Arthur Stillwell, of Moorcroft House, upon an insane patient, who had refused food, and had been fed by means of the stomach-tube, and whose case indeed suggested it. It succeeded.

In the case of poisoning, the *stomach-tube* itself must be used. Yet, frequently, patients make ineffectual efforts to vomit under the conjoint influence of an emetic and of a narcotic poison; if, in such a case, the stomach-tube be introduced, vomiting is frequently perfectly accomplished through the tube itself, and along its sides, from the influence of the tube on the excitors of vomiting, and, through them, on the cardia,—and the further operation of the syringe may be unnecessary. But cases of severe poisoning will, however, require the syringe both for the injection and the extraction of fluids into the stomach.

It is generally necessary in the insane to open the mouth, and to retain it open by force. To avoid this necessity, Dr. Hall proposes to introduce a smaller tube along *the floor of the nostril;* in doing this, we pass it *below* the nasal branches of the trifacial, which are the excitors of sneezing, and *above* the palatine branches of this nerve, which are the excitors of vomiting. This suggestion was also adopted by Mr. Arthur Stillwell. A long, narrow, flexible tube, of the diameter of a large male catheter, was employed. It was passed just seven inches through the nostril, being incurvated towards the pharynx. The patient appeared entirely unaffected by the procedure. The fluid food was then propelled along the tube. Deglutition took place regularly, marked by the usual movements of the larynx: in vain did the patient try to expel it; saliva only appeared at the lips; the food had passed beyond the domain of *volition;* it had been seized by the actions of the excito-motory power. It passed freely onwards into the stomach!

4. *Irritation of the Fauces, to induce vomiting.*—From an ignorance, observes our author, of the physiology of these parts, a singular event has occurred. A patient wishing to excite vomiting, has introduced a feather,—too far,—*beyond* the excitors of vomiting, which he ought to have stimulated to their proper action, *to* the excitors of deglutition, under the influence of which the feather has actually been swallowed.

Dr. Hall relates some cases in point. We are tempted to mention the manner in which, in two instances, a long feather has been extricated from the œsophagus. In a case which occurred to Dr. Cleghorne, the third or fourth feather of a goose-wing, whole and entire, had been swallowed. The Doctor, and Mr. Morris, a surgeon, furnished themselves "with a long flexible whalebone, to each extremity of which a piece of sponge was fixed, with two strings reaching betwixt each. The strings were added to the instrument by Mr. Tuckey, some years ago, when he had occasion to use it, that he might have it in his power to extract the sponge, in case it should break loose from the whalebone in the *œsophagus;* and we readily perceived they might be of singular service in the present case, by laying hold of the web, if the whalebone was turned round its *axis.*"

"We first tried, by depressing the root of the tongue and inspecting the *fauces,* if we could see any part of the feather, or reach it with our fingers; but, this being to no purpose, Mr. Morris introduced the smallest end of the abovementioned instrument into the left side of the *fauces,* to avoid pushing down the feather, which we had reason to think was lodged in the right side of the *œsophagus;* and, having thrust it far down towards the stomach, he began to make the extraction by slow degrees, inclining the end of the instrument he held towards the left side, and twisting it round repeatedly, that the strings might have a better chance to take hold of the feather.

This first attempt proved unsuccessful, and so did another which I made after the same manner; but the third time, Mr. Morris, having introduced the largest end of the instrument as far down as he could into the *œsophagus,* was lucky enough to bring up the feather along with it into the *fauces,* from whence he extracted it with his fingers, about two hours after it had been swallowed." 83.

5. *Intraction of the Rectum Bougie.*—These are by no means solitary cases of this kind. The other extremity of the alimentary tube presents us with a phenomenon of a similar character. The action of this part of the animal frame is highly peculiar: after the expulsion of its contents, the last intestine has, through the medium of the internal sphincter and the levator ani, an inverted action; and an instrument, introduced for the purpose of relieving the intestine, has been suddenly grasped, snatched from the fingers of the operator, and drawn up into the rectum and colon.

6. *Severe Pain in the Rectum and its Remedy.*—" There is a peculiar and severe pain of the rectum, which comes on in paroxysms, generally during the first sleep, and of which I have not seen any description in medical writings. It appears to me to depend on some hæmorrhoidal tumor, compressed by the action of the sphincter ani. The infallible remedy is—to rise out of bed, and to make a forcible and sustained effort to relieve the bowels. The changed position of the hæmorrhoid, under these circumstances of positively relaxed sphincter, and effort of protrusion, leads to perfect relief of the paroxysm of pain."

7. *Reduction of a Hæmorrhoidal Tumour.*—Dr. Hall observes that much difficulty has sometimes been experienced, not only by the patient, but by the surgeon, in returning protruding hæmorrhoids. Pressure alone is applied in vain. But if the patient be directed to make a forcible expulsive effort, and pressure be simultaneously made, the tumor frequently recedes immediately. The sphincter is positively relaxed; the ligature which it formed round the tumor is removed, and the reduction is easy. Dr. Hall asks: Would a similar measure aid the accoucheur in returning the prolapsed uterus? Would a similar effort ever assist in the passing of the male catheter?

8. *Intraction of the Female Catheter.*—The female catheter has been suddenly drawn out of the fingers of the surgeon into the cavity of the bladder. The accident is to be ascribed to successive, simultaneous contractions of the cervix vesicæ and levator ani, excited through incident and reflex nerves, in connexion with the spinal marrow.

### *Pathology of the True Spinal System.*

Dr. Hall observes that, in disease, the morbid stimulus may act upon the incident, the central, the reflex part of the excito-motory arc or arcs, and produce corresponding effects,—corresponding diseases. So we may have centripetal, or central epilepsy, and we may have spasmodic tic, and other affections, from an affection of the reflex or motor nerves. Dr. Hall divides the class of spasmodic diseases into three sub-classes, of which he presents a Table.

### Table of the Pathology of the True Spinal System.

I. *Diseases of the Incident Nerves.*

| | | | |
|---|---|---|---|
| I. | 1. *Dental*<br>2. *Gastric*<br>3. *Intestinal* | *Irritation in Infants.* | 1. *The Crowing Inspiration.*<br>2. *Strabismus, Spasm of the Fingers and Toes; Strangury; Tenesmus; &c.*<br>3. *Convulsion.*<br>4. *Paralysis.* |
| II. | 1. *Gastric*<br>2. *Intestinal*<br>3. *Uterine* | *Irritation in Adults.* | I. *Hysteria.*<br>II. *Asthma.*<br>III. *Vomiting; Hiccup; &c.*<br>IV. *Epilepsy,*<br>V. *Puerperal Convulsion; &c.* |

III. *Traumatic Tetanus; Hydrophobia: &c.*

II. *Diseases of the Spinal Marrow itself.*

I. *Inflammation and other Diseases.*

II. *Diseases of the Vertebræ and Membranes.*

III. *Counter-pressure, &c. in Diseases within the Cranium.*

IV. *Centric Epilepsy, Tetanus, &c.*

V. *Convulsions from Loss of Blood; &c.*

III. *Diseases of the Reflex or Motor Nerves.*

I. *Spasm.*
1. *Spasmodic Tic.*
2. *Torticollis.*
3. *Contracted Limbs; &c.*

II. *Paralysis.*

Dr. Hall observes that the undue or unduly diminished quantity of blood in the medulla oblongata induces spasm or paralysis. It has been found too, *in experiments*, that lesions of the encephalon induce *paralysis only*; whilst lesions of the medulla oblongata and spinalis induce *convulsion* or *paralysis*, according to its severity. Hence it becomes an important question to determine the cause of convulsive affections in *disease* of the encephalon. This cause appears to be either irritation or counter-pressure: the former may act through the medium of the nerves distributed to the membranes, as the recurrent of the trifacial of Arnold,—as in epilepsy induced by a spiculum of bone; the latter is illustrated by the case of meningitis by Dr. Abercrombie, already quoted, in which the anterior fontanelle became prominent; pressure upon it induced convulsion.

Dr. Hall proceeds to treat of some pathological facts in detail.

Disease, he says, may take place in any of the three divisions of which the true spinal or excito-motory system consists: in the course of the incident excitor nerves; in the spinal marrow or centre of the system; or in the reflex, motor nerves. The action of disease, in the first of these cases, is incident, and either direct in its course, or from *above, downwards*, or retrograde, that is, from *below, upwards*. It always induces a *spasmodic* affection. The action of disease, or of the causes of disease, in the third, is, he believes, never retrograde, and may induce either spasm, or paralysis.

1. *The Cynic Spasm.*—"The first fact which I shall adduce in illustration of these principles, is one to which I have ventured to apply the designation of the *cynic spasm;* for it is proably the σπασμος κυνικος of Hippocrates; and it is certainly allied to the Sardonic laugh. It is excited through filaments of the trifacial nerve (the '*nervi ethmoidalis ramus narium externus*,' the '*nervi lateralis narum superiores, et inferiores*'); these, when excited under particular circumstances, induce contractions of the levatores alæ nasi. In a patient in the deep coma of apoplexy, I pierced the skin on the cheek, the hand, the thigh, &c. with a pin; there was no manifestation of sensation—no motion whatever. I then touched the eye-lash and the *internal* nostril with a feather; this induced action of the orbicularis and levator alæ nasi: I then pricked the *exterior* part of the nostril with the pin; the action of the levatores was immediate."

2. *The Mechanism of Vomiting.*—Dr. Hall's idea is this:—The contents of the thorax and abdomen are subjected to the sudden and almost spasmodic contraction of all the muscles of expiration, the larynx being closed so that no air can escape from the chest, and the two cavities being made one by the floating or inert condition of the diaphragm.

The mere mechanism of the act of vomiting differs little, therefore, from that of coughing, by which, indeed, the contents of the stomach are frequently expelled. The larynx in the former is, however, permanently, in the latter only momentarily, closed; and there is, doubtless, a different condition of the cardiac orifice and of the œsophagus.

So far as we can see, there is no proof of that passive, or floating condition of the diaphragm which constitutes the gist of Dr. Hall's hypothesis. Experiments, observation, and actual feelings, rather go to establish the contraction of the diaphragm during the act. An inspiration is made, the diaphragm *remains down*, the larynx is closed, and the abdominal muscles at the same time contract. Vomiting is neither concurrent with inspiration nor with expiration. It follows the former, and precedes the latter. The breath in fact is held while we vomit. Of course the stomach may or may not contract. In many instances we believe that it does.

3. *Failure of the Excito-Motor Power.*—In cases of disease of the head, of exhaustion, of sinking—as the general powers of life decline, the gradual failure of the excito-motory power is particularly marked.

The first thing observed is, that the eye-lids do not perfectly close during sleep; they still, however, close on touching the eye-lash. In a short time, not only the continued action of the orbicularis is defective, but the excitability of the border of the eye-lid is impaired, and the orbicularis ceases to act on touching the eye-lash.

The second effect of a failing excito-motory power is an affection of the respiration. This is first *audible*, as in sleep. It then ceases to be *equable*, and at length consists in sudden, *catching* inspirations,—a state from which Dr. Hall has never seen a patient recover.

Lastly, the sphincters lose their power.

A Section on the *Therapeutics of the True Spinal System*, contains a few hints.

Their principal applications are in the treatment of asphyxia, of certain kinds of convulsion, of sickness, of asthma, of impaired power of the sphincter vesicæ.

In some cases of asphyxia, and especially in the case of the asphyxia of the newly-born infant, the excitement of the trifacial, or spinal, nerves, by exposure to the cold atmosphere, or dashing cold water on the face or trunk, is a most important remedy. We may also irritate the nostrils, the fauces, or the glottis, and attempt to excite the actions of sneezing, vomiting, and coughing. Galvanism may be applied with similar views.

The occurrence of convulsion is frequently prevented, and the larynx, closed in certain convulsions, is opened, by dashing cold water over the face.

Asthma is sometimes removed by the inhalation of certain vapours, as the smoke of the stramonium, the vapour of creosote, &c.

Enuresis has been remedied by the action of cantharides.

The feeble action of the uterus has been excited by the secale cornutum.

*The Ganglionic System, or System of Secretion, Nutrition, &c.*

Dr. Hall remarks that the cerebral system is concerned with *psychical* or mental acts merely; the true spinal, with *physical* acts on the *masses* of bodies to be appropriated to or expelled from the animal œconomy; whilst the ganglionic system relates to the *chemical* changes in the disposition of the *atoms* of the animal body, its solids, fluids, &c. He goes on to state that there are *external* as well as *internal* parts which require the interstitial deposit and re-absorption of the atoms of animal matter. It was to be *expected*, therefore, that there would be an external as well as internal ganglionic and nutrient system. This we find accordingly, although it has never been so viewed or stated before.

For the external organs, the ganglia on the posterior roots of the spinal nerves are probably destined; for the head, the series of ganglia found upon and in connexion with the trifacial nerve; for the lungs, stomach, &c. the pneumogastric; for the internal viscera, the ganglionic system, commonly so called.

Dr. Hall alludes in terms of just commendation to Sir Charles Bell's discovery of the difference of function between the anterior and posterior roots of the spinal nerves. He adds :—

"But there is no connexion between the function of sensation and the existence of a ganglion: the unequivocal sentient nerves, as the olfactory, the optic, the auditory, are without any thing very distinct of this kind, whilst the ganglionic nerves are without sensibility, or nearly so.

The questions, then, still remain, why are the portio major of the trifacial, especially, and of the posterior spinal nerves, provided with ganglia? The reply to these questions, and the argument, may be stated thus:

1. There is an internal nerve for formation, nutrition, secretion, &c. 2. This nerve is ganglionic. 3. There are external organs and structures requiring nutrition, &c. 4. There are also external ganglionic nerves. The inference is plain, that these constitute the external ganglionic sub-system. The fifth especially abounds with ganglia.

It is true that the semilunar and external spinal ganglia differ in appearance from the ganglia of the sympathetic, as Sir Charles Bell has well displayed. What is the nature of this difference? To this question I find no reply in authors. It is plain, however, that the difference consists in their being, alone, *plexic*. The internal ganglionic nerve is purely nutrient: its ganglia are simple. The external involve sentient, and I believe excitory, nerves, with the nutrient; they combine, therefore, the appearances of the plexus and of the ganglion.

But are there any other anatomical facts, any physiological and pathological facts, which lead us to think that the trifacial and spinal nerves, besides their other functions, possess a nutrient and secretory power? Indubitably there are.

The distribution of this nerve to the lacrymal, parotid, and submaxillary glands, can only be for secretion. I may refer, upon this point, to the beautiful Dissertation of Signor Bellingeri.

I must also notice particularly a branch of the trifacial nerve discovered by M. Arnold, and termed by him the *recurrent* of the fifth. It returns from the ophthalmic branch of this nerve, to be distributed between the laminæ of the tentorium. What can its function be, in this situation? It cannot be *touched*. It cannot, then, be for *sensibility*. It cannot be for *excited* motion. It can only be for formation, for nutrition. Such, then, is the *probable* function of this nerve." 122.

Dr. Hall sums up his observations on the Nervous System thus:

This system is divisible into—

I. The Cerebral, or psychical, or that of our relation to the external world *mentally;*

II. The True-spinal, or excito-motory, or that of the reflex action of the *vis nervosa*, or of our appropriation of certain objects of the external world *physically* and *in masses;* and

III. The Ganglionic, or the system of *interstitial*, or *chemical*, or *atomic* changes, of nutrition, secretion, &c. also under the immediate influence of the *vis nervosa*.

IV. Intermediate between the *first* and *second* of these is the influence of *emotion, passion*, &c.

V. Intermediate between the *second* and *third* are the internal muscular organs, the heart, the stomach and intestines, &c.: and these are under the *immediate* influence of the *vis nervosa* and the *irritability* of the muscular fibre.

VI. Lastly, the *tone* and *irritability* of the general muscular system are *constant* and *direct* effects of the True-spinal and Ganglionic systems, and of the *vis nervosa*.

The fourth Chapter is dedicated to the Fœtus and Infant. Some interesting remarks on the anencephalous fœtus are followed by others on the accidents or diseases of *birth—apoplexy* and *asphyxia*.

*Of Congenital Asphyxia.*—Dr. Hall draws particular attention to this.

"The infant is said to be *still-born*. We wait for the establishment of respiration, and this event does not take place. There is a general alarm. We now see the value and importance, in a *practical* point of view, of the principles of the physiology and pathology of the nervous system which I have been detailing. I have stated that respiration is an *excited* function; that it belongs to the excito-motory sub-division.

In one word, then, all our efforts must be instantly made to *excite* respiration. Now what are the channels through which this act may be excited? What are the excitors of respiration? The *trifacial*, the *pneumogastric*, and the *spinal* nerves.

The *trifacial* nerves must be excited by *forcibly* blowing or dashing cold water on the face, by stimulating the nostrils by ammonia, snuff, pepper, or the point of a needle.

The *spinal* nerves must be excited by *forcibly* dashing cold water on the thorax, the thighs; by tickling or stimulating the sides, the soles of the feet, the verge of the anus.

What the pneumogastric is, as the excitory nerve of respiration, under ordinary circumstances, the trifacial and the spinal nerves are, in cases of asphyxia, or suspended respiration. The means recommended for exciting respiration through these excitors, frequently induce a sudden act of inspiration, which proves the first of a series, so essential to animal life.

The important point to be mentioned, is, that it is not the mere application of cold, but the *sudden* application of *cold* to a *warm* surface, which is the effectual means of exciting respiration. It is the *sudden alternation*. To apply cold to a cold surface would only be to sink the general powers of life. The infant should be kept warm; the warm bath may be required; and then cold water must be applied, in *moderate quantity*, but with *force*.

But if these attempts to *excite* respiration through the trifacial and spinal nerves fail, we must *imitate* this function, by artificially distending the lungs, in the hope that, eventually, it may be excited through its wonted channel, the *pneumogastric*.

To effect this, the practitioner's lips are to be applied to those of the infant, interposing a fold of linen, and he is to propel the air from his own chest, slowly and gradually, into that of the infant, closing its nostrils, and gently pressing the trachea on the œsophagus. The chest is then to be pressed, to induce a full expiration, and allowed to expand, so as, if possible, to effect a degree of inspiration.

But it is important, in doing this, that the practitioner himself should previously make *several deep* and rapid respirations, and, finally, a full inspiration. In this manner, the air expelled from his lungs into those of the little patient, will contain more oxygen and less carbonic acid, and consequently be more capable of exciting the dying embers of life.

I base this suggestion on an interesting communication by Dr. Faraday, in the London and Edinburgh Philosophical Magazine, vol. iii, p. 241, for October 1833, to which I have already referred. It is ascertained that respiration may be suspended longer, as in diving, or in experiments, after such repeated forced respirations, than in ordinary circumstances, from the greater purity of the air in the lungs.

If all these plans should be tried in vain, I would strongly advise galvanic or electric shocks, to be passed from the side of the neck to the pit of the stomach, or in the course of any of the *motor respiratory* nerves, and their appropriate muscles. No time should be lost in sending for a proper apparatus; but, should the lapse of an hour, or even more, take place before it *can* be obtained, still it should be sent for and tried.

When respiration is established, the *face* must *still* be freely exposed to the air, whilst the temperature of the limbs and body is carefully sustained.

In the *midst* of these efforts, it should, in the next place, be the office of two other individuals to maintain or restore the *temperature* of the little infant, by gently but constantly pressing and rubbing its limbs between their warm hands, passing them upwards, in the direction of the venous circulation.

An enema of gruel, at 98° or 100°, or *higher*, with a little brandy, should be administered.

As soon as possible, a little warm liquid, as barley-water, at blood-heat, should be given by means of the proper bottle, furnished with leather or soft parchment. A teaspoon must not be used for fear of choking. If the infant draws the liquid through its own lips, by its own efforts, there is no danger.

Lastly, these various means should be continued or repeated in the most persevering manner." 142.

*Of Secondary Asphyxia.*—Dr. Hall directs attention to this.

When an infant has been restored from a state of asphyxia, it frequently relapses into a *secondary asphyxia*, and is lost.

Sir Humphrey Davy experienced a secondary attack after breathing hydro-carbonate. A corporal of the Guards, after being apparently restored from asphyxia from submersion, was affected with convulsions and expired.

In a case of asphyxia from laryngitis, after the patient had ceased to breathe, the trachea was opened, artificial respiration was performed, and re-animation took place; but the patient expired shortly afterwards.

These facts should keep us upon our guard against secondary asphyxia: we should watch our patient, and be prepared with all our remedies; we should dash cold water on the face occasionally, and expose the face of the patient to the cool, free, open air; and we should enjoin, in an adult, frequent, full, respirations.

Dr. Hall adds:—"This secondary asphyxia is the cause of *sudden* death in some other diseases, as the *crowing inspiration*, to be treated of hereafter. In this disease, the infant sometimes dies in an instant—in the twinkling of an eye—much more suddenly, in fact, than from *mere asphyxia*. I believe the blood is poisoned from the imperfect respiration, and that the *coronary circulation* becomes insufficient to sustain the action of the heart, and that this organ therefore fails. It is in this manner that the most sudden of sudden deaths occurs, both in some cases of what I have designated secondary asphyxia, and in disease of the heart itself, and especially in ossification of the coronary arteries,—the impeded circulation through them which occurs in the fatty heart, and other diseases of this organ and of its valves.

The *fact*, in regard to the crowing inspiration, should suggest what is found so useful in other respects in this disease; viz. through *change of air*, and free exposure to its genial influences. The same indication is especially to be fulfilled in *all* cases of asphyxia, for a considerable time after all apparant danger has ceased."

CHAPTER V. is occupied with the *Diseases of the Nervous System in Infants and Children.*

Some are of cerebral, some of spinal origin. The *cerebral* diseases of infants may be divided into—

1. *Encephalitis.*
2. *Tuberculous Hydrocephalus.*
3. *Hydrocephaloid Diseases.*
    a. *From Intestinal Disorder.*
    b. *From Exhaustion.*

Dr. Hall gives a good account of the symptoms of encephalitis and tuberculous hydrocephalus. The following case of supposed encephalitis, with anasarca, after Scarlatina, is interesting.

"A year and a half ago, Mr. Duffin took me to Highgate: we met Mr. Snow. The patient was a boy, aged twelve. Sixteen days before, he had gone through scarlatina, in its very mildest form; he had scarcely been confined to bed, and had not suffered from the *nimiâ medici diligentia;* he had appeared quite well.

On this Sunday morning he was seized with swelling of the face, which came on and increased equally suddenly and rapidly. With this symptom there was the appearance of sudden and serious collapse, and, soon afterwards, convulsions, followed by coma.

When I first saw my patient, there had been, first, the appearance of collapse and sinking, then convulsions, and these were followed by deep coma. Wine and brandy on the table indicated sufficiently the previous state of the case. I felt persuaded, in spite of these appearances, that the only hope was afforded by relieving the vascular system within the head, and yet that the measure was not unattended with danger. This view was freely explained to the boy's father, who very sensibly said he confided his son's life to the hands of his medical advisers.

We placed the patient upright, and Mr. Duffin opened the jugular vein. I kept my finger on the pulse whilst we allowed *twenty ounces* of blood to flow! The convulsions ceased, and the coma diminished, but did not disappear. We then ventured to cpen a vein in the arm, and abstracted seven more ounces of blood!

In less than an hour the little patient knew his parents. We prescribed calomel and purgative medicine; a cold lotion to the head, and fomentations to the feet: afterwards leeches were applied; but the bloodletting was *the* remedy to which the amendment was obviously due. The little boy recovered forthwith, and, what is important, without the least symptom of the morbid effects of loss of blood." 153.

Dr. Hall remarks: 1st, that acute anasarca and convulsions may, and frequently do, follow the mildest cases of scarlatina; perhaps, because, after *such* cases, less precaution is usually taken to clear the bowels; 2nd, that in all cases of acute anasarca there is the danger of an affection of the head, arachnitis, or congestion, coma, or convulsion; 3rd, that in such cases the remedy is bloodletting—*bloodletting until relief and security are obtained;* and 4th, that there is, in such cases, great *tolerance* of this remedy.

*The Hydrocephaloid Disease.*

Dr. Hall fairly vindicates his claim to originality in the description of this complaint.

The causes are thus mentioned. The exhaustion, on which it principally depends, has its origin, in early infancy, chiefly in diarrhœa, or catharsis; in the later periods of infancy, in the loss of blood, with or without the relaxed or evacuated condition of the bowels. The state of diarrhœa has generally depended upon improper food, or intestinal irritation. It has very frequently succeeded to weaning, or to other changes in the diet, or to constipation. The catharsis has followed the administration of an aperient medicine, which, at such a moment of disorder of the stomach and bowels, is apt to act excessively. The exhaustion from loss of blood generally follows the inappropriate or undue application of leeches, or the use of the lancet. Dr. Hall, indeed, gives an useful caution. Of the whole number, he says, of fatal cases of disease in infancy, a great proportion occur from this inappropriate or undue application of exhausting remedies. This observation may have a salutary effect in checking the ardour of many young practitioners, who are apt to think that if they have only bled, and purged, and given calomel enough, they have done their duty; when, in fact, in subduing a former, they have excited a new disease, which they have not understood, and which has led to the fatal result.

Dr. Hall divides the affection into two stages—the first that of irritability, the second that of torpor.

In the first stage, he goes on to observe, the infant becomes irritable, restless, feverish; the face flushed, the surface hot, and the pulse frequent; there is an undue sensitiveness of the nerves of feeling, and the little patient starts on being touched, or from any sudden noise; there are sighing and moaning during sleep, and screaming: the bowels are flatulent and loose, and the evacuations are mucous and disordered.

If, through an erroneous notion as to the nature of this affection, nourishment and cordials be not given, or if the diarrhœa continue, either spontaneously, or from the administration of medicine, the exhaustion which ensues is apt to lead to a very different train of symptoms. The countenance becomes pale, and the cheeks cool or cold; the eye-lids are half closed, the eyes are unfixed, and unattracted by any object placed before them, the pupils unmoved on the approach of light; the breathing, from being quick, becomes irregular and affected by sighs; the voice becomes husky, and there is sometimes a husky, teasing, cough; and, eventually, if the strength of the little patient continue to decline, there is a crepitus or rattling in the breathing; the evacuations are usually green; the feet are apt to be cold.

A similar train of symptoms occurs in other cases, in which the strength of the little patient has been subdued and the vascular system exhausted by the abstraction of blood. In both cases, leeches are sometimes again applied to subdue this new form of disease, under the erroneous notion of a primary cerebral affection. This measure infallibly plunges the little patient into imminent, if not irretrievable danger. Sometimes the sinking state goes on in spite of every appropriate remedy. Stimuli, if efficacious, reduce the frequency of the pulse, and restore the wonted warmth, colour, expression, and smiles to the countenance.

Dr. Hall particularly insists on a close observation of the condition of the cheeks, in regard to colour and warmth. That condition, he observes, may be considered as the pulse of very young infants, indicating the degree of remaining power, or of exhaustion. In the present case, especially, there is no symptom so important, so distinctive. It is from the condition of the cheeks, in conjunction with a due consideration of the *history*, that the diagnosis of this morbid state, and the indication of the appropriate remedies, are chiefly to be deduced. The general surface, and especially the hands and feet, also afford important sources of information as to the condition of the nervous or vital powers. Next to these, the degree of frequency of the pulse, and the character of the breathing, are points of the greatest importance; during the stage of irritability, the breathing is quick; during that of torpor, it is slower, irregular, suspirious, and, finally, crepitous; the pulse changes in its beat, from being full becoming smaller, but retaining, perhaps, its former frequency.

We should be especially upon our guard, not to mistake the stupor, or coma, into which the state of irritability is apt to subside, for the natural sleep, and for an indication of returning health. The pallor and coldness of the cheeks, the half-closed eye-lid, and the irregular breathing, will sufficiently distinguish the two cases.

The following is Dr. Hall's methodus medendi. The remedies, he says, for this morbid affection are such as will check the diarrhœa, and afterwards regulate the bowels, and restore and sustain the strength of the little patient. With the first objects, it may be necessary to give the tinctura opii, and chalk, and, afterwards, the pilula hydrargyri, rhubarb, and magnesia; with the second, sal volatile, but especially brandy; and proper nourishment, are to be given according to circumstances. But in this, as in many cases of infantile disorders, the milk of a young and healthy nurse is the remedy of most importance; in the absence of which, ass's milk may be tried, but certainly not with the same confident hope of benefit.

Five or ten drops of the sal volatile may be given every three or four hours; and twice or thrice in the interval, five or ten drops of brandy may be given in arrow-root done in water. As the diarrhœa and appearances of exhaustion subside, these remedies are to be subtracted, the bowels are to be watched and regulated, and the strength is to be continually sustained by the nurse's or ass's milk. The brandy has sometimes appeared to induce pain; sal volatile is then to be substituted for it; a dose of magnesia has also appeared to do good.

For the state of irritability, the warm bath is a remedy of great efficacy. For the coma, a small blister, or sinapism, should be applied to the nape of the neck. A state of exhaustion of the general system, as I have observed elsewhere, by no means precludes the possibility of real congestion of the brain. It rather implies it. In extreme cases, there are not only the symptoms of cerebral congestion during life, but effusion of serum into the ventricles of the brain is found on examination after death.

In every case, the extremities are to be kept warm by flannel, and the circulation should be promoted in them by assiduous frictions. It is of the utmost importance carefully to avoid putting the little patient into the erect posture. A free current of air is also a restorative of the greatest efficacy.

*The Croup-like Convulsion.*

Dr. Hall remarks that the origin of the croup-like convulsion was "erroneously referred" by the late Dr. J. Clarke to the cerebrum; and by the late Dr. Hugh Ley to compression and consequent paralysis of the pneumo-gastric and its recurrent nerves. It is in reality, he adds, an excitation of the true spinal or excito-motory system. It *originates* in

I. 1. *The Trifacial*, in teething.
   2. *The Pneumogastric*, in over- or improperly-fed infants;
   3. *The Spinal nerves*, in constipation, intestinal disorder, or catharsis. These act through the medium of

II. *The Spinal Marrow*, and

III. 1. *The Inferior* or *Recurrent Laryngeal*, the constrictor of the larynx;
   2. *The Intercostals* and *Diaphragmatic*, the motors of respiration.

*This* mode of viewing an important disease and *Class* of diseases is entirely new, and is the only true one. It points, too, to the *causes* and the *cure*. Dr. Hall presents us with an appropriate

*Table of the Crowing Inspiration.*

| I. *The Excitors.* | II. *The Centre.* | III. *The Motors.* |
|---|---|---|
| 1. *The Trifacial.* | *The Medulla oblongata.* | 1. *The Recurrent of the Pneumogastric.* |
| 2. *The Pneumogastric.* | | 2. *The Intercostals.* |
| 3. *The Spinal.* | | 3. *The Diaphragmatic.* |

When the *crowing inspiration* passes into actual *convulsion*, the larynx, from being *partially*, becomes *perfectly closed*, and there are violent *expiratory* efforts, with consequent congestion of the encephalon, and all its terrific train of evils!

The same mode of viewing this important subject leads us to give its proper place to *each* of the series of symptoms: the *spasmodic* or *spinal* are the *first* in order; the *cerebral* the *second*. We are thus enabled to see the just relation and position of effusion into the ventricles of the brain in regard to this disease; it is the *effect*, not the *cause*.

Dr. Hall first combats the opinion of *the cerebral origin* of the disease. He argues:—

1. That the *changes* in the symptoms, whether for better, or for worse, are far too sudden to be dependent on *disease* within the head.

2. That the *effects* of its causes and of its remedies are of a character totally different from what would be seen in such disease.

3. That hydrocephalus—tubercular hydrocephalus—does not commonly produce the croup-like convulsion. This statement must, however, be received with caution, and be submitted to new observation.

Dr. Hall next assails the hypothesis of Dr. Hugh Ley.

In the *first place*, he believes that the cases adduced to support this view, are not cases in point, but, in reality, cases of other diseases.

"*Secondly*, supposing pressure upon the pneumogastric to exist, it would induce totally different phenomena from those actually observed in this disease: and it would not explain the *series* of phenomena which actually occur in it; for,

Such pressure would induce simple *paralysis*. This would, in the first place, affect the recurrent nerves, and the dilator muscles of the larynx; it would induce a *partial* but *constant* closure of that orifice, and a permanent state of dyspnœa, such as occurred in the experiments of Legallois, or such as is observed to be excited in horses affected with "*cornage*" or *roaring*, as described by M. Dupuy in his treatise "De la Fluxion Périodique," 1829, p. 117, &c. It would also induce paralysis of the inferior portion of the pneumogastric, with congestion in the lung or lungs, and the well-known effects upon the stomach of the division of this nerve, with paralysis of the cardia.

The disease in question, on the contrary, variously designated, "*peculiar convulsion*," "spasm of the glottis," &c., is obviously a *part* of a more general spasmodic affection, and frequently, indeed most frequently, comes on in the midst of the first *sleep*, in the most *sudden* manner; receding equally *suddenly*, to return, perhaps, as before, after various intervals of days, weeks, or even months; very unlike paralysis from *any* cause. Nay, the convulsive efforts in the muscles about the larynx are frequently quite obvious. There is even opisthotonos, or comprosthotonos, in some cases.

*Thirdly*. It not unfrequently involves or accompanies, as I have said, *other* affections *indisputably spasmodic*, as distortion of the face, strabismus, contractions of the thumbs to the palms of the hands,—of the wrists, feet, toes, general convulsions, and sudden dissolution,—a series of phenomena totally unallied to paralysis.

*Fourthly*. Indeed the larynx is sometimes *absolutely closed*,—an effect which *paralysis* of the recurrent nerve and of the dilator muscles *cannot* produce.

*Fifthly*. Paralysis from the pressure of diseased glands would be a far less *curable disease*, a far less *less variable* disease, a far *less suddenly fatal* disease than the croup-like convulsion.

*Sixthly*. Almost all recent cases are at once relieved by attention to three or four things, viz. the state, 1, of the *teeth*; 2, of the *diet*; 3, of the *bowels*; and 4, by change of *air*; they are as obviously produced or reproduced by the agency of errors in one or more of these.

*Seventhly*. In fact, the croup-like convulsion is a *spasmodic* disease, excited by causes situated in the nervous centres, or eccentrically from them; in a case of spina bifida already mentioned, a croupy and convulsive inspiration was induced by gentle pressure on the spinal tumour; in cases from teething the attack has been induced and removed many times by *teething*, and by freely *lancing the teeth*, by crudities, and by emetics, by constipation and by purgatives, by change of air, &c.

*Eighthly*. There is a series of facts which prove the connexion of this disease with other forms of convulsions in children, and with epilepsy in the adult subject.

*Ninthly*. In protracted cases, congestion and effusion within the head occur as *effects* of this disease.

*Lastly*. Innumerable cases of undoubted croup-like convulsion have occurred, in which no enlarged glands could be detected in any part of the course of the pneumogastric nerve."

Dr. Hall's idea of treatment comprises the remedies—

1. *Against the attacks*.
2. *In the attacks, and in the threatening of the attack*.
3. *Against their effects*.

The remedies against the attacks, or the prevention, consists in avoiding all the exciting causes: dental, gastric, intestinal irritation; passion; vexation; disturbance; interrupted sleep, &c.

The remedies in the threatening of attacks consist in the watchful and prompt repetition of the same treatment; lancing the gums, relieving the stomach and the bowels. The *sleep* especially should be watched, and if there be a sardonic smile, or starting, or other symptoms, the little patient must be *gently* awakened, and the remedies just enumerated should be administered. Mental agitation must be most cautiously avoided. After the gum-lancet, Dr. H. would advise a copious enema of warm water.

If there be great threatening of an attack, Dr. Hall would tickle the fauces, dash cold water on the face, and irritate the nostrils, having the patient placed, as speedily as may be, in the warm bath.

To guard against the effects of the attacks, we may deplete the blood-vessels about the head with cupping, or leeches, apply an alcoholic lotion constantly all over the head, or, if the case be urgent, the ice-cap.

In addition to these measures, the secretions must be corrected, mild mercurials being given, perhaps, to affect the system; and diuretics, if the urine be scanty; afterwards, change of air is of undoubted efficacy; and a very mild tonic plan may be added with advantage, as minute doses of the sulphate of quinine, of the carbonate of iron, &c. Sponging with *warm* salt and water is also a valuable auxiliary remedy.

Dr. Hall dilates very fully on the employment of the *gum-lancet*. He does not use it merely to let the teeth traverse the gum, but as a sort of depletory scarification. We should lance, says he, the gums *freely* and *deeply*, over a great part of their extent, *daily* or *even twice a day*, and apply a sponge with warm water, so as to encourage the flow of blood.

The principal part to be lanced is the more prominent portion of the gum; but, in some urgent instances, Dr. H. has recommended the *lateral* portions also to be freely scarified.

We fear if this plan were generally adopted, that much mischief would be inflicted on the teeth.

Dr. Hall, after some further remarks, adds:—" I cannot conscientiously omit the expression of my conviction that the bloodletting system, and the calomel system of treatment, recently in vogue in this complaint, under the sanction of the highest authority, and under the impression that the affection was of *cerebral origin*, have been extremely injurious in their consequences.

I have seen only one case in which the plan which I have here proposed has proved unsuccessful, when employed in the *dawn* of the disease; and that case was attended by induration of the medulla oblongata, and tetanic symptoms; it was, in a word, of centric origin. But I have seen innumerable cases in which bloodletting and calomel had not prevented the recurrence of convulsions, and the consequent effusion into the ventricles of the brain."

For our own parts, while we admit that there is a great deal of truth in Dr. Hall's observations, we must say that they appear to us to be rather too exclusive. We certainly do think that, in some instances, it is requisite to attack the head more actively than he advises. At the same time, we would earnestly beg the practitioner to bear what is said by our author in mind.

*Paralysis from Dentition.*

The following is an interesting case, the more so as a child of Dr. Grant's of Thayer Street, was the subject.

"A little girl, aged twenty mouths, was taken, when suffering feom dentition, with loss of the power of elevating the right arm—that of closing the hand remaining; there was no *other* symptom of cerebral affection. The suffering from dentition was undoubted; I therefore concluded that the case was one of paralysis from teething.

The gums were freely lanced, the bowels were well moved, the diet strictly regulated; and, for *fear* of hidden disease within the head, two leeches were applied. An embrocation was prescribed for the arm.

A few days after the attack of paralysis, this little girl was seized, in the early part of the night, with a fit of crowing inspiration. This event confirmed *me* in my diagnosis. The issue justified the view I had taken. The child recovered *perfectly*, without any energetic remedy being used for cerebral affection, by continued attention to the state of the gums, the stomach, and the bowels; an event which could scarcely have occurred from such simple measures, had there been such decided affections arising from cerebral disease." 189.

The sixth chapter is devoted to the—

*Pathology of the Nervous System in Adult Age.*

From infantile to adult age the *cerebral* system undergoes a gradual and progressive development; many of the phenomena of the *true spinal* system are consequently *obscured*. But they are only obscured, for, in the close of life, the true spinal functions are as energetic and as essential as its dawn.

Dr. Hall again draws attention to the distinction between *centric* and *eccentric* forms of disease of the nervous system—a distinction of the greatest importance, no doubt. Some observations on the leading characters of *cerebral* and of the *true spinal diseases* merit notice.

*Cerebral* diseases, says Dr. Hall, affect primarily the cerebral functions, and the true spinal functions consecutively. The diseases of the true spinal system induce changes in the excito-motory phenomena in the first place, and in the cerebral functions in the second. Cerebral diseases are generally more insidious in their progress than the true spinal, because slight aberrations of the cerebral functions are less observable than similar affections of the true spinal system; pain, vertigo, watchfulness, &c. are less striking than the slightest degree of convulsive movement or paralytic affection. It is on this account that the *first* symptom *observed* in cerebral disease is frequently one belonging to the true spinal system, especially *vomiting*, perhaps *strabismus*.

The true spinal diseases, especially those of eccentric origin, affect, in a remarkable manner, the set of functions belonging to this system: those of the eye and eye-lid; those of the larynx, of the pharynx; the respiration; the action of the expulsors and of the sphincters; that state of the muscles designated by the term *tone*. We must revolve in our minds the symptoms of epilepsy, of hysteria, of tetanus, of hydrophobia; the causes and phenomena of vomiting, of asthma, of abortion, of tenesmus, and of strangury, and we shall be forcibly struck wirh the justice of this remark. On the other hand, every convulsive effort affects the brain with congestion and its consequences, of which a fatal coma or effusion are not the least frequent.

Memoirs on the Condition of the Muscular Irritability in Paralytic Limbs —on the Morbid Reflex and Retrograde Actions of the Spinal Marrow— and on the Distinct Influence of Volition, of Emotion, and of the Vis Nervosa, follow. These Memoirs were published in the Medico-Chirurgical Transactions, in reviewing which, we noticed them fully. It will not be necessary, therefore, to revert to them.

The seventh chapter is occupied with the *Diseases of the Nervous System in Adult Age*.

Dr. Hall treats first of the:—

### *Diseases of the Cerebral System.*

Speaking of *Encephalitis*, Dr. Hall notices a fact which should be recollected. From insensibility, the patient does not void the bladder; this viscus becomes excessively distended, and there may be a stillicidium urinæ. In *every* case of insensibility—in every case of involuntary discharges of urine, we must examine the hypogastric region. We may observe that the same thing is apt to occur in fever, when there is cerebral oppression.

Dr. Hall draws attention to another practical fact. Not only the dawn and the course of encephalitis are insidious, but its termination is particularly so. In some cases, an unexpected state of *sinking* takes place, in which the symptoms, whether pain or delirium, &c. subside, and the patient is thought to be convalescent. The same event occurs in some other diseases, especially enteritis. We must beware of this fact, too, and suspect some such insidious change, unless *all* the symptoms concur to denote returning health.

While alluding to the morbid appearances of encephalitis, Dr. Hall particularly directs attention to the fact—that the inflamed brain is *tumefied*. This fact explains the occurrence of pressure, and its varied effects on different parts of the encephalon, frequently situated remotely from the part affected with inflammation, softening, &c. It is on this principle that we explain the occurrence of various affections of the true spinal system in inflammation of different parts of the cerebral system,—the strabismus, the vomiting, the various convulsions which occur in the early stage; and the stertor, the relaxed sphincters, &c. which occur in the later stages of encephalitis.

*On Congestion and Hæmorrhage.*

Dr. Hall occupies a section with the subject of cerebral congestion and cerebral hæmorrhage. He observes, and observes truly, that the *antecedent* symptoms of palsy and apoplexy, like those of many other diseases, can only be learnt in private practice. In fact, however beneficial observation in hospitals may be, a man would be a coarse and a careless, and an unsuccessful practitioner who acted upon it alone.

Dr. Hall remarks that the attack of congestion, or of hæmorrhage itself, is frequently of the most sudden kind. There is total loss of sense and motion; the patient is flushed, comatose, breathes with stertor, and the pulse is strong and full. This is probably the case of congestion. In the attack of considerable hæmorrhage, there are the symptoms of *shock* inflicted upon the nervous system; pain of the head is followed by paleness, sickness, and vomiting, and fainting; coma, or paralysis, loss of speech, or of the power of swallowing, succeed immediately, or more slowly, probably according to the promptitude or the extent of the hæmorrhage. In the attack of partial hæmorrhage, these symptoms are observed in a slighter form; and it is some paralysis, hemiplegia, partial loss of speech, &c. which ensues. These observations, though, perhaps, in the main correct, are certainly not universally true. We have seen cases of congestion attended with symptoms of depression, and of hæmorrhage, with such symptoms as are attributed to congestion.

Dr. Hall applies his views with regard to bloodletting to the treatment of these cases.

"The principal point which I wish to press upon my reader, in reference to bloodletting, is its different measure proper in mere congestion and actual rupture. In the former there is extreme tolerance of loss of blood; in the latter, the system is extremely, and even dangerously, susceptible of this loss. The diagnosis is frequently difficult. I have pointed out the most distinctive symptoms. In addition to an attention to these, I must repeat the important injunctions—to place *the patient* in the perfectly upright posture before the blood is allowed to flow; to watch his countenance, his breathing; to keep *the finger* on his pulse; and, the moment the slightest indication of approaching syncope takes place, to arrest the flow of blood and place the patient recumbent.

If early syncope occur, we must trust the future to local depletion by means of cupping to the occiput and the neck. If the patient lose a large quantity of blood without change, pursue and repeat the remedy boldly; his life depends upon ample depletion of the sanguiferous system: we must add to the energetic use of the lancet, that of the cupping instrument." 278.

There follow some remarks *on the Use of Tracheotomy.*

This may be employed so as to afford *time* for remedies to act. Dr. Physick suggested it for hydrophobia—it *might* be employed in that form of tetanus in which the patient dies of asphyxia—but, says Dr. Hall, "in *apoplexy from congestion,* in the *coma after epilepsy, in puerperal convulsion,* I am persuaded that the fatal event might be averted by the timely adoption of this measure. The patient dies of asphyxia, and of an asphyxia which tracheotomy would, I believe, prevent; or of coma, which blood-letting would cure!" Dr. Hall adds—"there *is* such a case as paralysis of the pneumo-gastric nerve, and of the dilator muscles of the larynx; it occurs in deep intoxication, and probably in other cases of coma, as in that of apoplexy, of epilepsy, from opium, &c.

A patient affected with such paralysis from deep intoxication was rescued from impending death by tracheotomy, at the suggestion of Mr. Sampson of Salisbury. The case is published in the Medico-Chirurgical Transactions, vol. xx, p. 45, and forms one of the *most splendid achievements of modern surgery.*"

After narrating the case at length, Dr. Hall concludes—it must be remembered—1. that dashing cold water on the face opens the larynx, and induces inspiration; 2. that there is one other measure to which recourse should be had more frequently: it is the induction of vomiting by irritating its excitors in the fauces; 3. that these and other measures must of course be fully tried before we proceed to the more formidable remedy of tracheotomy; 4. but that tracheotomy is a most important resource, and to be instituted when—*the others fail*, and *before it is too late.*

*Mode of Action of the Causes affecting the Nervous System.*—There are some brief but useful remarks on this head. It may be stated, says Dr. Hall, as a general principle, that the *first* effect of injury done to the nervous system, is a diminution of its functions; whilst the second or ulterior effect is the augmentation of those functions.

If we divide the spinal marrow in the frog, there is at the first a cessation of the reflex actions with that of the circulation; by-and-by, these return; after a time, their force is morbidly augmented.

In injuries of the brain and of the spinal marrow, in man, the same series of phenomena is observed.

When the trifacial nerve is injured by exposure to cold, there is at first numbness; afterwards, perhaps, painful tic.

When the facial nerve is affected by severe cold, there is, at first, *paralysis,* and the face is drawn to the opposite side; afterwards there is *spasm,* and the face is drawn to the side affected.

In other instances, the first effect of the disease or injury subsides, without any subsequent augmentation of function. In hemiplegia, the paralysis of the face, the lips, the tongue, the leg, the arm, generally becomes mitigated, in the order enumerated, as the disease subsides, or is mitigated by our remedies.

In like manner the other diseases enumerated may pass from the first stage into a state of mitigation or recovery, without passing into the second stage.

On the other hand, the symptoms of the first stage may go on augmenting until death ensue, or the loss of the use of the limb or part affected be complete.

*Effects of Disease of the Cerebellum.*

Dr. Hall is inclined to think that disease of the cerebellum induces its peculiar effects on the genital organs, by irritating the medulla oblongata. The effects observed in strangulation, in some cases of epilepsy, as well as in several experiments, induce him to suppose so.

Vomiting sometimes occurs as a prominent symptom, as in many other diseases of the encephalon. This, as well as the affections of the genital organs, is obviously a result of irritation of the medulla.

Convulsions are more frequent in diseases of the cerebellum than paralysis. They affect many parts, and resemble epilepsy; or only one part. There can be little doubt that it is the adjacent medulla oblongata which is really irritated so as to produce these phenomena.

In some instances there has been a loss of balance, such as occurs in intoxication.

Sometimes the sensibility has been affected,—exalted or impaired. In some cases there has been amaurosis.

Diseases of the cerebellum, when they induce paralysis, usually affect the *opposite* side of the body, and the inferior more than the superior extremities.

*Tubercles in the Encephalon.*—Dr. Hall makes an observation in regard to the diagnosis of these morbid growths, which, though it refers to no new fact, is still perhaps not familiar to all.

"In speaking of tubercles of the brain, I must allude to the important law discovered by M. Louis, in regard to tubercles:—that, beyond the age of fifteen, tubercles are never found in *any organ* of the body without being present in the *lungs*? In a doubtful case, then, we examine the condition of the thorax: if there be tubercles there, it is a presumption that there may be tubercles in the encephalon; if there be no sign of pulmonary tubercle, it is a presumption that the affection of the brain is not tuberculous. But, as pulmonary tubercles are not always easily detectible, we endeavour to ascertain, in the absence of signs, whether there be other reasons for suspecting tuberculous formation,—such as in hereditary strumous or tuberculous disposition, the general or local signs of tuberculous affection in the system, or in any organ, &c." 288.

### *Mania.*

Dr. Hall asks:—"Are the morbid appearances *cause* or *effect* of the mania? That they may be the effect, and that it has been too exclusively regarded as the cause, I can scarcely doubt. The appearances are usually deposits of serum and of lymph between the arachnoid and pia mater; sometimes effusion into the ventricles, sometimes injection of the cortical substance.

If encephalitis is the frequent effect of mental harass and effort, why may not these appearances be the effect of the maniacal state?" Of course, the question is an important one, incapable, at present, of determination. But it is worth keeping in mind.

Dr. Hall has other views to propose. He inquires, 1. May not the pathology of mania be entirely the result of *shock* to the nervous system, arising from *mental effort,* in one case, and *mental emotion* in the other? And, 2, may not that pathology be entirely *intra-vascular,* as in the case of *exhaustion* from loss of blood, undue lactation, &c.

### *Non-restraint of Lunatics.*

We agree with Dr. Hall in the following opinion. It has recently been proposed to abandon *every kind* of restraint. But this is to reject necessary resources for the safety, and remedies for the malady, of the patient.

What is required is, kind and judicious restraint, in the hands of kind and judicious guardians.

But *the* all-important means of cure consists in *society* in which *decorum* and *gentleness* of conduct are *strictly enforced*.

### *Diseases of the Cerebral Nerves.*

There is a section on this subject. The following case of repeated amputations on account of pain in the stump is interesting. It is communicated to our author by Mr. Bransby Cooper.

*Case.*—" In June, 1834, a young married woman was admitted into Guy's Hospital with an 'irritable stump,' having submitted to three amputations under the following circumstances. In the first place, she fell across a fender, and produced such extensive injury to the wrist joint as to render it necessary to apply to a hospital for relief, and, I believe, was first admitted into the London Hospital, under the care of Mr. John Scott, who amputated the forearm above the wrist. Of this operation she recovered in the usual time, and was discharged as cured. In a few weeks, however, the stump became very irritable, and she suffered from neuralgic pains, which became so intolerable that she applied to St. Thomas's Hospital, when she was admitted under the care of Mr. Tyrrell. (I am not sure whether she was at St. Thomas's or the London Hospital first.) A second amputation was then performed,—still, I think, below the elbow,—when the same circumstances followed as after the former operation, perhaps with somewhat of an increase of neuralgic sufferings, when she fell under the care of Mr. Langstaff, who, after trying constitutional remedies, had recourse to a third amputation, above the elbow. In June, 1834, the stump of the upper arm giving the same suffering as she had before been subjected to, she presented herself at Guy's Hospital, still willing to submit to any treatment which could offer any hope of permanent relief: and, after trying veratria, stramonium, belladonna both internally and externally, without affording any mitigation of her sufferings, she readily submitted to the only remaining means which afforded a prospect of success—amputation at the shoulder joint, which she bore with heroic fortitude, and described the suffering as being infinitely less than she had frequently experienced during the paroxysms of her complaint; nor, in the division of the trunks of the nerves, did there appear to be more pain inflicted than in amputations under ordinary circumstances. The wound readily healed, nor had she ever after any severe neuralgic symptoms, although for some time there seemed rather more irritability about the surface of the skin than usual. In about two months after the amputation, she left the hospital, cured. I have several times seen this patient since; the last time about three years since, when she had had no return of her former distressing symptoms." 303.

As an instance of the *influence* of the trifacial nerve upon *vision*, Dr. Hall mentions a case under his own care. In it a partial *amaurosis* of the right eye has arisen, apparently from caries of the upper canine tooth of the right side; it was augmented by unsuccessful attempts at extraction; it has not ceased, however, since the extraction was effected.

Dr. Hall relates a case of paralysis of the trifacial nerve in its exterior course, reminding his readers, at the same time, of the difference between the cause of paralysis of sensation of the face in hemiplegia, or from division of the *trifacial* in a part of its course *exterior* to the cranium, and that arising from the division or disorganization of this nerve *within* the cranium. In the first two cases the eye is unaffected, in the last, this organ gradually perishes, he supposes, from the destruction of its ganglionic or nutritive portion.

*Paralysis of the Hypoglossal Nerve.*—This case is quoted from Dupuytren. There were rheumatism of the neck, situated along the vertebræ and occiput; and the gradual loss of muscular power, with atrophy of one half of the tongue, the sense of taste towards the base of the tongue remaining entire. The myo-glossal nerve is supposed to have been involved in the disease at and after its exit from the cranium; hence the affection of the tongue. The scarificator and cupping-glasses were repeatedly applied behind the mastoid processes, with satisfactory results.

*Paralysis of Motion from Pressure on the Anterior Roots of the Spinal Nerves.*—The case has been depicted and described by Sir Charles Bell in the Philosophical Transactions, for 1840.

"Plate IX. exhibits a tumor which engaged the *anterior* roots of the several nerves as they form the *cauda equina* within the tube of the vertebræ. The tumour was of the form of an almond, but larger, and into it the *motor* roots, both of the right and left side, were gathered, whilst the *sensitive* roots were free." "The lower extremities were deprived of motion, whilst they retained sensibility, and symptoms of so mnch interest were noted and discussed by the physicians and pupils." 313.

Dr. Hall doubts whether *spasm* ever affects the true cerebral nerves. He says:—The substance of the brain; the olfactory nerves; the retina, the optic nerve; the auditory nerve; the glossopharyngeal, are *insensible* and *inexcitor* when wounded or pinched. Wounds of these do not induce spasmodic contraction. No experiment has hitherto been made upon any purely *cerebral* voluntary nerve, with the view of determining whether, in such a case, there would be spasmodic action. Perhaps such a nerve does not exist entirely free from the intermixture of *true spinal* filaments. Is the levator palpebræ of this character? These, with many other questions, are still left for future inquiry,—by experiment and observation.

### *Diseases of the True Spinal System.*

Such diseases, says Dr. Hall, of the spinal marrow, as may materially affect its functions, induce, in the first place, paralysis of the cerebral nerves, sentient and voluntary, which run along its course, forming a part of its structure; and, in the second, either an excited or paralysed condition of its own peculiar functions. The symptoms combine, therefore, paralysis of sensation and voluntary motion, in parts below the disease, with spasm and ultimately paralysis, resulting from the affection of the *true* spinal marrow. Dr. Hall notices these diseases in the following order:

I. *The Central Diseases, or Diseases of the True Spinal Marrow itself.*
II. *The Centripetal Diseases, or Diseases excited through the Excitor Nerves.*
III. *The Centrifugal Diseases, or Diseases of the Motor Nerves.*

*Inflammation within the Spine.*—Dr. Hall acknowledges the difficulty of distinguishing, by the *symptoms,* inflammation of the meninges from that of the substance of the spinal marrow. But, in general, the symptoms of meningitis are *more* those of *irritation* of the spinal marrow, or *spasm*; those of *myelitis*, *more* those of *destruction* of the organ, or paralysis. Both kinds of symptoms may exist, however, or follow each other, in both diseases.

Dr. Hall thus proceeds to specify the symptoms as well as he can.

Amongst the first symptoms of spinal *meningitis* is local *pain* in some part of the spinal column, augmented by the movements of the patient, and by percussion, but rarely, if ever, by pressure along the spine. This pain sometimes extends along the back and limbs, in which there is then tenderness on pressure,—a symptom which may serve to distinguish meningitis from myelitis, in which there is usually loss of sensibility.

The next important symptom is spasm, or various kinds of muscular contraction. The head, the neck, or the trunk is bent backward, or there is trismus, torticollis, partial or complete opisthotonos, or contraction of the limbs, constant, or recurrent, or exacerbated, in paroxysms, on moving, on being moved, &c. with extreme pain; sometimes there are convulsions.

The respiration is sometimes difficult. There is sometimes retention of urine and constipation.

The symptoms will vary according as the meningitis exists at the base of the brain, at the upper, or at the lower, part of the spine principally.

The symptoms of spinal *myelitis* are those of paralysis of sensation and voluntary motion; a sense of numbness, an impaired sensibility, a sense of feebleness, an impaired muscular power, are first observed, singly or combined, in one or both of the inferior or superior extremities.

In some cases, probably of complication with meningitis, there is augmented sensibility. In other cases there are spasmodic or convulsive affections.

If the disease proceeds, the paralysis of sensation and voluntary motion gradually augments. Generally the paralysis affects first the inferior, and afterwards the superior extremities; far more rarely it pursues a contrary course: occasionally the motions alone, and very rarely the sensations alone, are paralysed.

If the disease occupy the *upper* parts of the spinal marrow, the respiration, and even the action of the larynx and pharynx become impaired, and we have difficulty or choking in swallowing, or asphyxia. There is sometimes the sensation of a cord-like tightness across the epigastrium. If the *lower* part of the spine be affected, the bladder, the rectum, and their sphincters, are variously paralysed, and there may be retention of urine and constipation, or involuntary evacuations, or retention and involuntary flow of urine may be combined. The condition of the bladder and the condition of the rectum should be ascertained by proper examinations, *in every case.*

In some instances there is perfect impotence, or inertia of the uterus: in others, the patient has become a father, or the uterus has been excited to expel the fœtus.

Dr. Hall passes to *the Centripetal Diseases*, which originate at a distance from the spinal centres. And first of *Centripetal Epilepsy.*

This form of epilepsy takes its origin in the *excitor nerves* of the true spinal system, involving the axis of this system, and its motor nerves, in their turn; functionally, however, not organically. It is to be viewed as *curable*, however *difficult* of cure. By avoiding the exciting causes, its attacks are avoided; the susceptibility to returns subsides; these returns become less frequent and less severe and at length frequently cease altogether.

The principal causes of eccentric epilepsy are—1, the presence of indigestible food in the *stomach;* 2, the presence of morbid matters in the *intestines;* 3, *uterine* irritation. The first of these acts through the medium of the pneumogastric; the second and third through that of peculiar spinal nerves,—all *excitors* belonging to the true spinal system.

Dr. Hall presents a table of the phenomena of epilepsy.

TABLE OF EPILEPSY.

| I. *The Excitors.* | II. *The Centre.* | III. *The Motors.* |
|---|---|---|
| 1. *The Pneumogastric in the Stomach.* | *The Medulla Oblongata.* | 1. *The Recurrent of the Pneumogastric, closing the Larynx.* |
| 2. *The Spinal in* | | 2. *The Spinal, inducing* |
| 1. *The Intestine.* | | 1. *Forcible Expirations* |
| 2. *The Uterus.* | | 2. *Convulsion.* |
| | | 3. *Expulsion of the Urine, &c.* |

The treatment of this form of epilepsy essentially consists in avoiding the causes, in moderating the paroxysms, and in subduing vascular fulness. The strictest rules must be laid down for the diet, for the state of the bowels, for conducting the catamenial periods. These last should be passed in bed; the feet and abdomen should be fomented; the warm-water enema, warmer than usual, and the opiate enema, should be administered.

The immediate accession of the paroxysm may sometimes be prevented by dashing cold water on the face, or by exciting the nostrils by snuff, &c. In this manner the disposition to closure of the larynx, and expiratory efforts, is exchanged for sudden acts of inspiration. Or the fauces must be irritated, or an efficient emetic be given.

The stupor, or coma, continues Dr. Hall, induced by the paroxysm, may require the administration of bloodletting, general or topical, according to its degree and deviation, and probable effects. But *tracheotomy* is the remedy against the stupor, or coma, following the attack of epilepsy, if it exist in an *extreme* degree.

"The views which I have given of centripetal epilepsy are amply confirmed by the facts, that there is no constant morbid change observable in this disease, and that many patients, after long years of its attacks, have finally and fully recovered; facts which ought to encourage us steadily to pursue the mode of treatment." 326.

We must say that this quite accords with our own experience. Many cases of epilepsy are remediable, if not curable. The great thing is to avoid the exciting causes, and to determine, if it be possible, on what the epilepsy depends.

Dr. Hall thus distinguishes hysteria from epilepsy;—vast indeed, he observes, is the distance which separates hysteria from epilepsy; yet how *similar* are the symptoms of these two diseases. There is *one* great distinction—in hysteria, much as the larynx may be affected, it is never closed; in epilepsy it is closed: in the former, we have *heaving, sighing, inspiration;* in the latter, *violent, ineffectual efforts,* at *expiration:* in the former, the cerebrum, the true spinal marrow, are comparatively unaffected; in the latter, they are in a state of congestion.

Dr. Hall naturally laughs at the idea of animal magnetism.

*Spasmodic Asthma.*

The following passage will shew what Dr. Hall's views are:—"The simplest form of asthma is that immediately induced by taking some indigestible substance; or, perhaps, still more immediately induced by the inhalation of certain kinds of dust diffused in the atmosphere, as that raised by shaking a feather-bed, the powder of ipecacuanha, &c. The incident branches of the pneumogastric, or internal excito-motory nerve, are excited; the action is reflected by the medulla oblongata upon the motor branches, and, as I believe, upon the circular muscular fibres of the bronchial tubes. These tubes are contracted, and the phenomena of asthma are induced. A constipated state of the large intestine acts in the same manner through the incident spinal nerves. Contracted bronchial tubes explain all the phenomena: the dyspnœa, the urgent, rapid, imperfect, bronchial inspiration; the protracted wheezing bronchial expiration; the bronchial rattles under the stethoscope, especially during expiration; the excited secretion of mucus; the cough; ultimately, the dilated air-cells, the dilated heart, &c."

*Tenesmus and Strangury.*

"I had recently a most interesting case of spasmodic stricture of the sphincter ani. The finger could scarcely be introduced. It was discovered that a calculus existed in the urethra; when this was removed the stricture immediately ceased." 339.

Tenesmus almost always implies the existence of strangury, and strangury that of tenesmus. They induce each other; they are *similar excito-motory* phenomena, affecting the exits of two organs.

*Spasmodic Strabismus.*

"I have already noticed the strabismus which arises from *paralysis* of cerebral and voluntary nerves, and some of the muscles of the eye-ball. I now wish to draw my reader's attention to another form of strabismus, not hitherto distinguished from the former, and which I believe to be an affection of the roots of the motor nerves of the true spinal system.

In the former case, the patient can frequently move the eye-ball fully, in every direction except one; at a certain point, the eye-ball stops, although the other eye continues to pursue an object placed and moved before it. This is the case with a patient at this moment under my care for attacks of sickness, with defective vision and motions of the eye.

In spasmodic strabismus the motions of the eye may be perfect, except on certain occasions of excitement, or of disorder, or of intense application, or of employment of the eye; the strabismus then becomes apparent; the eye-ball is obviously *drawn* in one particular direction.

In one interesting little girl, aged about three years, the strabismus came on whenever a stranger came into the room, whenever she was asked to read, &c." 342.

The last Chapter is dedicated to:—

*Diseases of the Nervous System of Remote Origin.*

They are enumerated thus,

I. *Intestinal Irritation.*
II. *Exhaustion from loss of blood.*
III. *Chlorosis.*
IV. *Arthritis.*
V. *Excessive study, shock, alcohol, &c.*
  1. *Delirium tremens.*
  2. *Delirium traumaticum.*
VI. *Affections of the kidney.*
  1. *Dropsy.*
  2. *Diabetes.*
  3. *Other morbid affections of the Urine.*
  4. *Ischuria.*

*Intestinal Irritation.*—This consists of the irritation of indigestible food, scybala, or other morbid contents of the stomach, or bowels, excited into activity by some *shock* of the system, or of the nervous system, such as a fall or other accident, parturition, &c.

The symptoms are, rigor, frequently severe heat of surface, and violent pain of the head, with intolerance of light and of sound; the symptoms, in a word, of the most acute encephalitis.

The breath is tainted, the tongue loaded and swollen, the secretions morbid. The first step to be taken in a doubtful case, is very slowly to administer an enema of from three to three and a half pints of warm water, and to examine the state of the fæces, and observe the effect upon the disease and upon the system. If there be scybala, if the symptoms be subdued, and especially if there be faintishness, the case is indubitably not cerebral inflammation, but intestinal irritation.

If the case still remain doubtful, we must prepare the arm, open a vein, and then place the patient upright, and let the blood flow until the lips become pallid; if the case be encephalitis, an extreme quantity of blood will flow, even thirty or forty ounces, or more, before there is any appearance of syncope; if it be intestinal irritation, syncope occurs before one-fourth of that quantity of blood has left the circulating system!

We believe we have selected many observations of interest and value. We have been guided in our choice by a desire to present what is most novel, and most important. Repetition there must be, for that is a characteristic of Dr. Hall's works.

Of the volume itself we need *say* little, having *done* so much. It is a fair sample of Dr. Hall's efforts, and would stamp him, were there nothing else to do so, as a keen and vigorous thinker, and a man of originality and genius. We trust he will continue to devote his talents to the prosecution of the physiology and pathology of the nervous system.

